# Supplementary material for: Electroacoustic tomography for real-time visualization of electrical field dynamics in deep tissue during electroporation
Source: Commun Eng. 2023 Oct 23;2:75. doi: 10.1038/s44172-023-00125-9 (PMC10955875; doi:10.1038/s44172-023-00125-9)
Supplement: Supplementary file 2 — Supplementary Information [file 44172_2023_125_MOESM2_ESM.pdf]

Supplementary Information

Electroacoustic Tomography for Real-time Visualization of Electrical Field Dynamics in Deep Tissue during Electroporation

Yifei Xu[1], Leshan Sun[1], Siqi Wang[1], Yuchen Yan[1], Prabodh Pandey[2], Vitalij Novickij[3,4*],

Liangzhong Xiang[1,2,5 *]

[1]*The Department of Biomedical Engineering, University of California, Irvine, CA, USA*

[2]*The Department of Radiological Sciences, University of California at Irvine, Irvine, CA, USA*

[3] *Institute of High Magnetic Fields, Vilnius Gediminas Technical University, Vilnius, Lithuania*

[4] *Department of Immunology, State Research Institute Centre for Innovative Medicine, Santariškių 5, 08410, Vilnius, Lithuania*

[5] *Beckman Laser Institute & Medical Clinic, University of California, Irvine, Irvine, CA, USA*

[*] co-corresponding author: liangzhx@uci.edu

[*] co-corresponding author: vitalij.novickij@vilniustech.lt

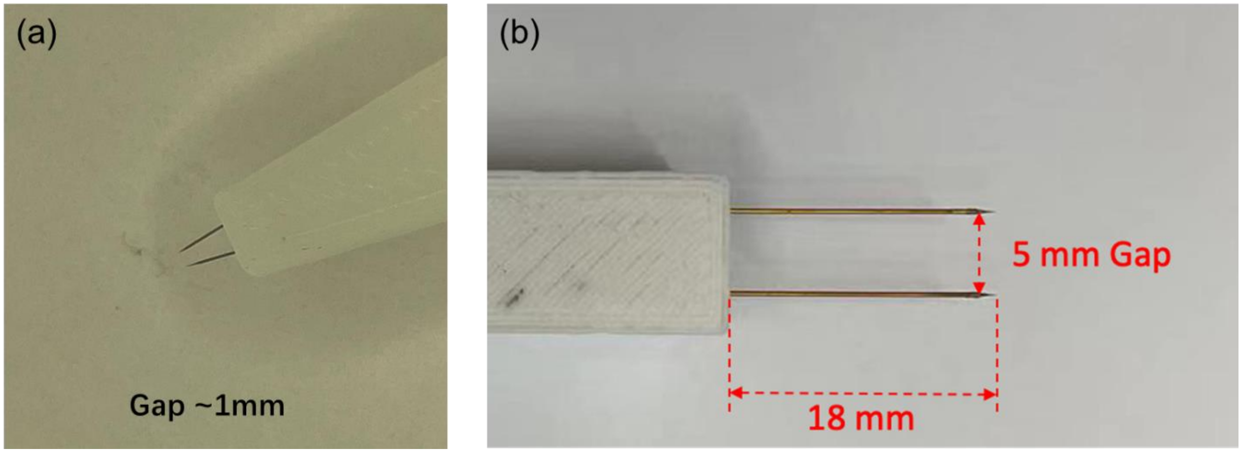

Supplementary Fig. S1 | Electrodes used in EAT systems. (a) Electrode diameter is 0.005' and spacing is set to 1 mm (b) Electrode diameter is 0.01" and spacing is 5 mm.

Supplementary Note. S1: The electrodes are fixed by a 3D-printed holder. Fig. S1(a) shows electrodes with 0.005'' diameter and ~1mm gap, used for high-intensity pulsed electric field generation. And Fig. S1(b) shows electrodes with 0.01'' diameter and 5mm gap. The larger electrode spacing helps us distinguish each electrode's location in EAT images.

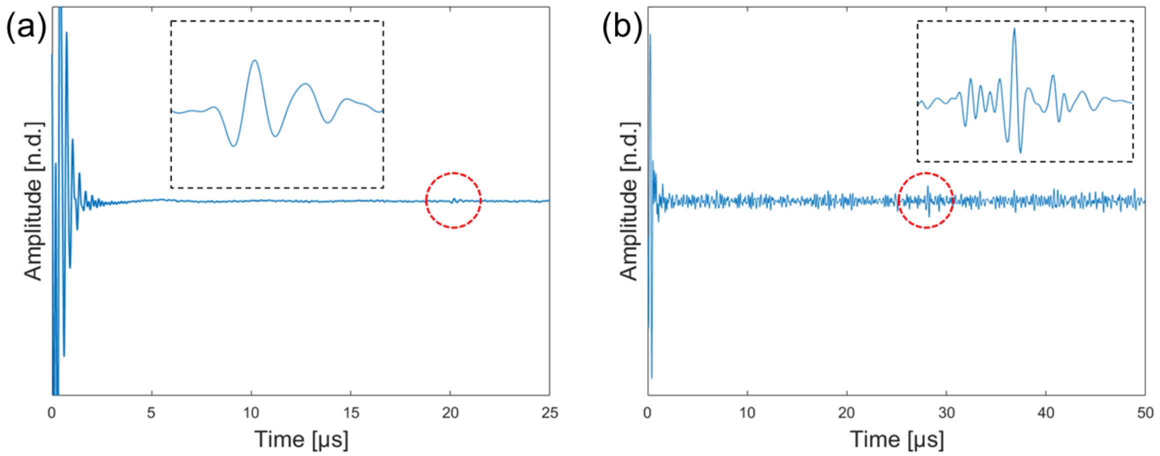

Supplementary Fig. S2 | Sensitivity testing of the EAT system. (a) using a single-element ultrasound transducer, and (b) using a ring-array ultrasound transducer. Partial zoom in the dashed box.

Supplementary Note. S2: The sensitivity of the two systems used in this system is demonstrated. We gradually reduce the applied voltage until the generated electroacoustic signal cannot be distinguished. For the single-point probe system (Fig. S2(a)), the electrodes are placed at a distance of ~3 cm from the transducer, and the minimum voltage that can be detected is 30 V, corresponding to an electric field intensity of 60 V/cm. For the ring array system (Fig. S2(b)), the electrodes are placed in the center of the array, about 5 cm from the transducer unit, and are able to detect a minimum voltage of 40 V, corresponding to an electric field intensity of 80 V/cm. This difference is influenced by the electrode-to-transducer distance but is mainly due to the size of the transducer unit.

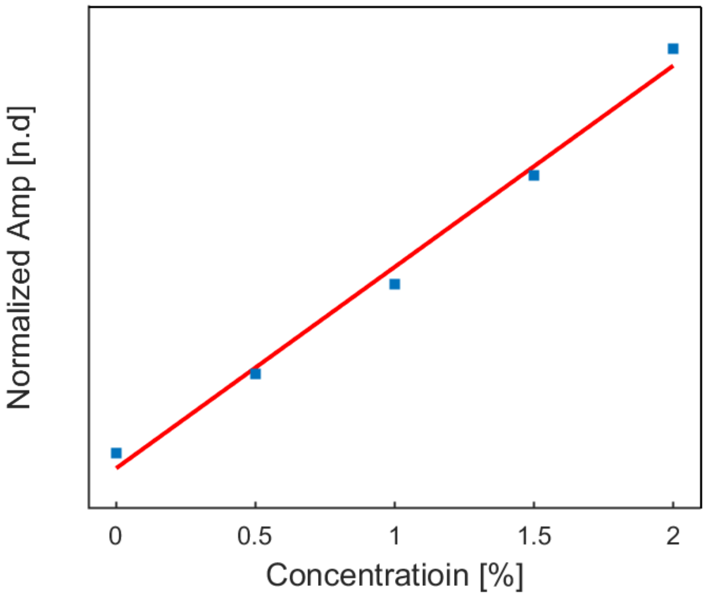

Supplementary Fig. S3 | Relationship between electroacoustic signal amplitude and phantom ion concentration. The amplitude characteristics of the electroacoustic signal are positively and linearly correlated with the ion concentration of the agar block phantom.

Supplementary Note. S3: Saline agar phantoms with different concentrations of edible salt were used to test how ion concentration affects the electroacoustic signal. Each block contained 3% agar powder, and five concentrations ranging from 0% to 2% were made with a 0.5% gradient for ionic concentration gradient experiments. And a linear relationship between EA signal amplitude and ion concentration was demonstrated.

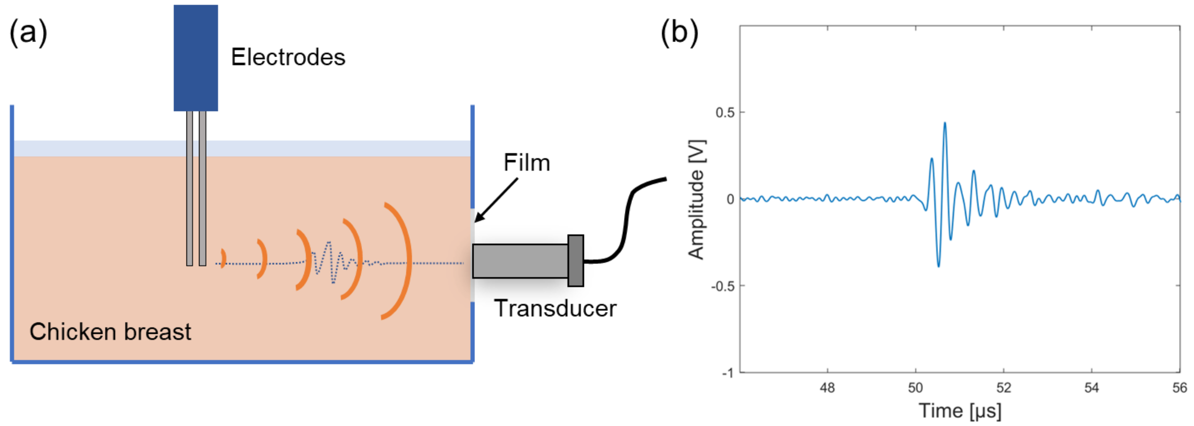

Supplementary Fig. S4 | EAT signal propagation dept. (a) schematic diagram of the depth testing system. (b) EAT signal was detected at a depth of 7.5 cm.

Supplementary Note. S4: The setup used for signal propagation depth testing is shown in Fig. S4(a). Fresh chicken breasts purchased from the supermarket were sliced and filled in the water tank, and the gaps were filled with deionized water. The side walls of the water tank were perforated and covered with polyethylene film, and the ultrasonic transducer was coupled to the film outside the water tank via ultrasonic gel to receive the electroacoustic signal. The electrode was placed at a distance of 7.5 cm from the transducer, and a voltage of 1 kV was applied to generate an electric field of 2 kV/cm. The detected ultrasound signal is shown in Fig. S4(b), where the signal is processed by a simple band-pass filter but not averaged. This experiment demonstrates that the EAT signal has a propagation depth of greater than 7.5 cm in biological tissues.
