# Supplementary material for: Electroacoustic tomography for real-time visualization of electrical field dynamics in deep tissue during electroporation
Source: Commun Eng. 2023 Oct 23;2:75. doi: 10.1038/s44172-023-00125-9 (PMC10955875; doi:10.1038/s44172-023-00125-9)
Supplement: Supplementary file 3 — Description of Additional Supplementary Files [file 44172_2023_125_MOESM3_ESM.docx]

**Description of Additional Supplementary Movie**

**Supplementary Movie S1 | Real-time visualizing electrical field dynamics with different pulse voltages.** EAT image of the same sample as Fig.5. The voltage applied to the electrodes was increased from 100 V to 1100 V. The position of the two electrodes and the gradually expanding range of electric field energy deposited around the electrodes were observed.

**Supplementary Movie S2 | Real-time visualizing electrical field dynamics with different electrode locations.** EAT image of the same sample as Fig.6. The electrodes are moved from the edge to the center of the ultrasound ring array. Real-time changes in the location of electrical energy deposition are observed. The energy of the electrical pulses remains constant in the experiment, but a gradually increasing range of signals is observed. This is because the signal was captured by more transducer elements while the electrodes moved.
